# A model to predict delivery time following induction of labor at term with a dinoprostone vaginal insert: a retrospective study

**DOI:** 10.1007/s11845-023-03568-3

**Published:** 2023-11-10

**Authors:** Fenghua Huang, Huijun Chen, Xuechun Wu, Jiafu Li, Juanjuan Guo, Xiaoqin Zhang, Yuan Qiao

**Affiliations:** Department of Gynecology and Obstetrics, Zhongnan Hospital of Wuhan University, Wuhan, 430071 Hubei China

**Keywords:** Dinoprostone, Full-term pregnancy, Induction of labor, Predictive model

## Abstract

**Background:**

Dinoprostone vaginal insert is the most common pharmacological method for induction of labor (IOL); however, studies on assessing the time to vaginal delivery (DT) following dinoprostone administration are limited.

**Aims:**

We sought to identify the primary factors influencing DT in women from central China, at or beyond term, who underwent IOL with dinoprostone vaginal inserts.

**Methods:**

In this retrospective observational study, we analyzed the data of 1562 women at 37 weeks 0 days to 41 weeks 6 days of gestation who underwent dinoprostone-induced labor between January 1^st^, 2019, and December 31^st^, 2021. The outcomes of interest were vaginal or cesarean delivery and factors influencing DT, including maternal complications and neonatal characteristics.

**Results:**

Among the enrolled women, 71% (1109/1562) delivered vaginally, with median DT of 740.50 min (interquartile range 443.25 to 1264.50 min). Of the remaining 29% (453/1562), who delivered by cesarean section, 11.9% (54/453) were multiparous. Multiple linear regression analysis showed that multiparity, advanced maternal age, fetal macrosomia, premature rupture of membranes (PROM), and daytime insertion of dinoprostone were the factors that significantly influenced DT. Time to vaginal delivery increased with advanced maternal age and fetal macrosomia and decreased with multiparity, PROM, and daytime insertion of dinoprostone. A mathematical model was developed to integrate these factors for predicting DT: *Y* = 804.478 − 125.284 × multiparity + 765.637 × advanced maternal age + 411.511 × fetal macrosomia-593.358 × daytime insertion of dinoprostone − 125.284 × PROM.

**Conclusions:**

Our findings may help obstetricians estimate the DT before placing a dinoprostone insert, which may improve patient management in busy maternity wards and minimize potential risks.

## Introduction

Induction of labor (IOL) is defined as the stimulation of uterine contractions to start labor by medications or other methods. The indications for IOL are mainly related to maternal or fetal medical causes and sociocultural preferences [1]. The global rise of IOL rates makes it important to ensure that IOL is both safe and reliable [2, 3]. A previous randomized trial compared IOL with expectant management at 39 weeks among low-risk nulliparous women revealed that IOL in women at term rarely result in adverse perinatal outcomes and reduced the incidence of cesarean delivery [4].

In China, prostaglandin E2 (PGE2) is the most common pharmacological agent used for IOL. Dinoprostone, which is a vaginal insert containing PGE2, exerts its effects locally by inducing cervical ripening and increasing the sensitivity of the uterine myometrium to oxytocin, thereby improving the rate of successful vaginal delivery [5]. Studies have shown that the risk of neonatal mortality is higher for night-time delivery, as compared to daytime delivery, especially in busy maternity wards [6]. Consequently, differences in time of delivery are associated with differences in the maternal and neonatal risks encountered in the wards. Moreover, some have shown that the DT following IOL with PGE2 was different for morning and evening deliveries [7]. However, due to the differences in ethnic background of patients and methods of obstetric management, data in China are limited on the prediction of the DT following PGE2-induced labor on the basis of the indications of placement time, parity, and maternal age and so on.

In order to address these knowledge gaps, we sought to conduct a historical study to identify the dominant factors influencing DT and to further use these data to develop a model that would allow prediction of the DT at or beyond term for each woman.

## Methods

### Participants

Data of pregnant women at or beyond term who had received a 10-mg dinoprostone vaginal insert for IOL between January 1^st^, 2019, and December 31^st^, 2021, at our institution were screened for eligibility. We included women who delivered a live singleton fetus in the vertex presentation at 37 weeks 0 day to 41 weeks 6 days of gestation had no contraindication to vaginal delivery and had not planned elective cesarean delivery. Data on gestational age were considered reliable if the woman was certain of the date of her last menstrual period and that date was consistent with the results of ultrasonography performed before 21 weeks 0 day or if the woman was uncertain of the date of the last menstrual period but the results were available from ultrasonography performed before 13 weeks 0 day. The exclusion criteria were as follows: (1) fetal anomalies, (2) cervical conization or cervical cerclage for incompetence, (3) a scarred uterus, and (4) allergies to PGE2. Data were collected on the maternal age, parity, body mass index (BMI), time of placement of dinoprostone insert (day or night), and indication for IOL. The time of the insert placement was classified as daytime (between 6 a.m. and 6 p.m.) or night-time (between 6 p.m. and 6 a.m.) placement. Additionally, all relevant maternal and fetal demographic data as well as data on obstetric complications and delivery were collected. The DT was calculated from the time of vaginal insert placement to the birth time.

### Procedure and management strategy

Women at or beyond term who consented for IOL were assessed again by the obstetrician-in-charge or above to reconfirm the indications for IOL. Before IOL, the cervix was examined to assess cervical dilation, cervical effacement, consistency, fetal position, and fetal station and to calculate the modified Bishop score.

The dinoprostone vaginal insert commercially available in China under the name Propess (10 mg, Ferring, Saint-Prex, Switzerland) was used in this study. The insert was placed in the posterior vaginal fornix to stimulate cervical ripening. Cervical conditions were considered to be unfavorable for dinoprostone use if patients had a modified Bishop score of ≤ 6, including those with the following obstetric complications such as intrauterine growth restriction (IUGR), fetal macrosomia, premature rupture of membranes (PROM), oligohydramnios, late-term pregnancy, and gestational diabetes or hypertension. After placement of the vaginal insert, the women were required to stay in the supine position for 30 min and fetal heart rate (FHR) was monitored for 2 h. Pelvic examinations were performed only at the start of active labor or in cases of complication. The insert was left in place until the start of labor or for more than 24 h.

### Trial outcomes

The start of labor was defined by the occurrence of frequent uterine contractions recorded over a 30-s period, along with cervical changes. The insert was removed when uterine hypertonus or hyperkinesia led to FHR anomalies, and a utero-relaxant agent was given as quickly as possible. In the case of inadequate uterine contractions or failure to progress after removal of the insert, labor was induced with oxytocin perfusion and amniotomy after 30 min of the removal. Delivery via cesarean section half-way was defined as IOL failure. The indications for cesarean delivery included stagnation in the active labor for 6 h from the onset of oxytocin perfusion or amniotomy, fetal distress, excessive vaginal bleeding, or social preferences.

### Statistical analysis

Continuous data are presented as mean ± standard deviation (SD) or median (interquartile range). Categorical data are presented as number (percentage). The Kruskal–Wallis and Wilcoxon Mann–Whitney tests were used to compare outcomes of women with different characteristics. Additionally, multivariate linear regression analysis was performed to identify the factors that may be associated with DT by Durbin-Watson and analysis of variance (ANOVA) tests. A *P* value of < 0.05 was considered to be statistically significant. All analyses were performed using SPSS version 24.0 for Windows (SPSS Inc., Chicago Illinois USA).

## Results

### Characteristics of the participants

**Fig. 1:**
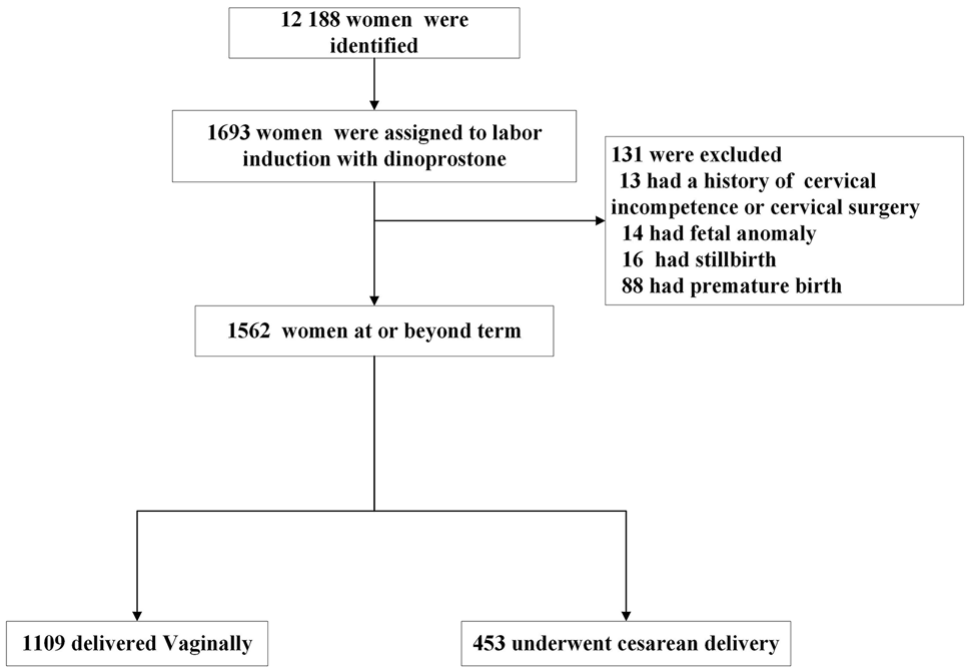
**Eligibility, delivery, and assessment.** Delivery with dinoprostone was defined from 37 weeks 0 day to 41 weeks 6 days

A total of 12,188 women who were managed at our center between January 1^st^, 2019 and December 31^st^, 2021 were screened. Data were collected for 1693 of them who underwent IOL with dinoprostone insertion. After excluding 131 patients who did not meet the inclusion criteria for our study, 1562 (92%) women at or beyond term who consented to IOL with dinoprostone were included in this study: 1109 (71%) of them delivered vaginally, while 453 (29%) delivered by cesarean section (Fig. 1).

First, the indications for IOL in the analyzed cases are summarized in Table 1. PROM (25.2%) (394/1562) and fetal macrosomia (21.1%) (330/1562) were the indications for IOL in nearly half of the cases, followed by gestational diabetes (17.2%) (268/1562) and late-term pregnancy (15.3%) (239/1562). The other indications, in descending order of frequency were hypertension (8.1%) (126/1562) and IUGR (0.4%) (7/1562). Among the women who had a cesarean delivery, approximately 88.1% were nulliparous and the remaining 11.9% were multiparous. The major indications for cesarean section following the placement of the dinoprostone vaginal insert were arrested active phase (23.6%), fetal distress (23.2%), and failed IOL (19.6%) (Table 1).

**Table 1:** Obstetric complications and outcomes of women with dinoprostone-induced labor

	**Induction by dinoprostone insert (*N* = 1562)**
**Indications of IOL**	
Late-term pregnancy	239 (15.3)
PROM	394 (25.2)
IUGR	7 (0.4)
Fetal macrosomia	330 (21.1)
Oligohydramnios	146 (9.3)
Gestational diabetes	268 (17.2)
Hypertension	126 (8.1)
Others	52 (3.3)
**Delivery route**	
Vaginal delivery	998 (90.0)
Operative vaginal delivery	111 (10.0)
**Cesarean delivery**	
Nulliparity	399 (88.1)
Multiparity	54 (11.9)
**Cesarean indications**	
Fetal distress	105 (23.2)
Failed induction of labor	89 (19.6)
Arrested active phase	107 (23.6)
Others	152 (33.6)

Data expressed as number (percentage)

Furthermore, we analyzed the maternal and neonatal baseline characteristics of the 1109 women who delivered vaginally (Table 2). The median maternal age in this group was 30 years (interquartile range 28–32 years). The median gestational age at which labor was induced was 40 weeks (interquartile range 39–40 weeks). In addition, the median BMI of the women was 25.5 (interquartile range 22.3–29.6), with 88.1% of them having a BMI of < 25. The mean fetal weight was 3360.84 ± 367.468 g. The time of placement of the dinoprostone insert was daytime for 632 women (57%) and night-time for 477 women (43%).

**Table 2:** Maternal and neonatal characteristics of vaginal delivery with dinoprostone-induced labor

	**Vaginal delivery (*n* = 1109)**
**Characteristic**	
**Age (y)**	
Median	30
Interquartile range	28–32
Age < 30 y	472 (42.6)
Age 30–34 y	514 (46.3)
Age ≥ 35 y	123 (11.1)
**Gestation (weeks)**	
Median	40
Interquartile range	39–40
< 39	204 (18.4)
39–40 + 6	847 (76.4)
≥ 41	58 (5.2)
**Parity**	
Nulliparity	898 (81.0)
Multiparity	211 (19.0)
**Abortion**	
Yes	337 (30.4)
No	772 (69.6)
**BMI**	
Median	25.5
Interquartile range	22.3–29.6
BMI < 25 (%)	977 (88.1)
BMI ≥ 25(%)	132 (11.9)
**Fetal weight (grams)**	3360.84 ± 367.468
< 2500	8 (0.7)
≥ 4000	33 (3.0)
**Time of dinoprostone insertion**	
Day	632 (57.0)
Night	477 (43.0)
**APGAR score < 7**	
1 min	7 (0.6)
5 min	0 (0)

Body mass index (BMI) was defined as the weight in kilograms divided by the square of the height in meters. Data are expressed as mean ± standard deviation (SD), or the median (interquartile range) or number (percentage)

### Primary influencing factors for vaginal delivery

All variables that could have influenced the DT are listed in Table 3. The median DT was 740.50 min, with an interquartile range from 443.25 to 1264.50 min. The Kruskal–Wallis test showed that women with advanced maternal age of ≥ 35 years had a longer median DT than those with age of < 30 years and those between 31 and 34 years of age (621.50 min (interquartile range 459.00–952.75 min) vs. 561.00 min (interquartile range 323.00–1085.00 min) or 1582.00 min (interquartile range, 1342.00–1582.00 min); *H* = 72.715; *P* = 0.000). In addition, no differences in the DT were noted between women aged less than 30 years and those aged 31–34 years (*Z* = 0.944, *P* = 0.345). Furthermore, median DT was significantly shorter for multiparous women than for nulliparous women (1282.50 min (interquartile range 541.00–1678.00 min) vs. 667.00 min (interquartile range 434.25–1084.25 min) *Z* = 3.44, *P* = 0.001). Additionally, daytime dinoprostone insertion led to a shorter median DT than night-time dinoprostone insertion (447.50 min (interquartile range 312.00–580.00 min) vs. 1207.50 min (interquartile range 920.00–1577.25 min, *Z* = 11.738, *P* = 0.000). Moreover, women with PROM also had a shorter median DT than those without (555.00 min (interquartile range 427.00–1004.00 min) vs. 874.00 min (interquartile range 483.50–1297.00 min); *Z* = 2.431, *P* = 0.015), and presence of fetal macrosomia was associated with a significantly longer median DT than its absence (1450.00 min (interquartile range 1320.75–2029.00 min) vs. 717.50 min (interquartile range 434.75–1187.25 min); *Z* = 3.823, *P* = 0.000). No statistically significant difference in the median DT was noted in the case of BMI and other obstetric complications, including late-term pregnancy, gestational diabetes, oligohydramnios, and hypertension.

**Table 3:** The time of vaginal delivery by dinoprostone-induced labor

	**Time to delivery (min)** ***M* (P25, P75)**	**Estimated difference** **Confidence interval** **(95%)**	***Z*/*H*-value**	***P* value**
**All vaginal deliveries**	740.50 (443.25, 1264.50)			
**Age (y)**			72.715	0.000
< 30	621.50 (459.00, 952.75)			
30–34	561.00 (323.00, 1085.00)			
≥ 35	1582.00 (1342.00, 1582.00) *			
**Parity**		−364.00 (− 577.00 to − 149.00)	3.44	0.001
Nulliparity	1282.50 (541.00, 1678.00)			
Multiparity	667.00 (434.25, 1084.25)			
**BMI**		−56.00 (− 194.00 to − 75.00)	0.820	0.412
< 25	676.50 (420.25, 1161.25)			
≥ 25	760.50 (454.75, 1290.75)			
**Time of dinoprostone insertion**		734.00 (643.00–826.00)	11.738	0.000
Day	447.50 (312.00, 580.00)			
Night	1207.50 (920.00, 1577.25)			
**Indications of IOL**				
**Late-term pregnancy**		−16.00 (− 149.00 to 111.00)	0.263	0.792
Yes	816 (436.00, 1179.50)			
No	680 (456.00, 1280,00)			
**PROM**		161.00 (30.00–306.00)	2.431	0.015
Yes	555.00 (427.00, 1004.00)			
No	874.00 (483.50, 1297.00)			
**Fetal macrosomia**		−802.00 (− 1083.00 to − 462.00)	3.823	0.000
Yes	1450.00 (1320.75, 2029.00)			
No	717.50 (434.75, 1187.25)			
**Gestational diabetes**		−144.500 (− 339.00 to 21.00)	1.699	0.089
Yes	983.50 (490.25, 1542.25)			
No	722.00 (435.00, 1197.75)			
**Oligohydramnios**		61.50 (− 152.00 to 266.00)	0.634	0.526
Yes	743.50 (401.75, 1286.25)			
No	740.50 (465.75, 1264.50)			
**Hypertension**		−143.00 (− 398.00 to 83.00)	1.301	0.193
Yes	840.00 (525.00, 1660.00)			
No	738.00 (434.50, 1260.00)			

Data are expressed as the median (interquartile range)

^*^Versus < 30 or 31–34, *P* < 0.05

Subsequent multiple linear regression analysis showed that advanced maternal age of ≥ 35 years, multiparity, fetal macrosomia, daytime placement of dinoprostone insert, and PROM were factors that significantly affected DT following IOL with dinoprostone (Table 4). The regression equation was given as follows: *Y* = 804.478 − 125.284 × multiparity + 765.637 × advanced maternal age + 411.511 × fetal macrosomia − 593.358 × daytime placement of dinoprostone insert − 125.284 × PROM.

**Table 4:** Prediction model for time to delivery

Model		Coefficients		Standardized coefficients	t**	Significance	Confidence interval (95%)	
		B*	Standard error	Beta***			Inferior limit	Superior limit
	Variable	804.478	186.108		4.323	0.000	437.847	1171.108
	Multiparous	−125.284	50.165	−0.097	−2.497	0.050	−224.107	−2.461
	Age ≥ 35 y	765.637	86.541	0.479	8.847	0.000	595.154	936.120
	Fetal macrosomia	411.511	114.942	0.139	3.580	0.000	595.154	934.120
	Daytime insertion	−593.358	49.509	−0.506	−11.985	0.000	−690.889	−495.826
	PROM	−125.284	50.165	−0.097	−2.497	0.013	−224.107	−26.461

^*^“*B*” represents the effect of each variable on DT; the + or − sign indicates the type of impact

^**^“*t*” refers to the significance level of B

^***^“Beta” refers to the change in the SD for DT for an increment of one SD of the explanatory variable when all other variables are constant

## Discussion

In this study, we investigated a large sample of 1693 pregnancies to identify the factors related to DT following use of dinoprostone inserts and thereby develop a model for prediction of DT in Chinese women. In 2021, the Chinese government introduced a series of law amendments to boost the birth rate [8]. Dinoprostone is a proven and effective agent used widely in clinics to induce cervical ripening agent for IOL. Improving the rate of vaginal delivery and ensuring safety are concerns that need to be addressed regarding dinoprostone use for IOL. Vaginal delivery may depend on various factors, including race/ethnicity, socioeconomic status, age, and medical conditions [9, 10]. 

In our study, the median DT was 740.50 min (12 h 33 min). Our findings are different from those reported in previous observational studies. A previous trial that predicted the mean DT with a dinoprostone insert in France reported a mean DT of 1239 min (20 h 39 min) [11]. The study included 405 patients, and the major factors influencing DT were found to be parity, BMI of ≥ 25, cervical dilation, and PROM. The difference in DT between ours and the French study can be due to several reasons. Compared with the gestational age of 34–42 weeks in their study, the gestational age in our study was at 37 weeks to 41 weeks 6 days. Furthermore, more than 80% of the women in our study had a BMI of < 25. Additionally, there may be racial differences between the French and Chinese populations. On the other hand, the rate of cesarean delivery after intravaginal dinoprostone insertion in our study (29%) is similar to that reported in another study (23%) from Hubei [12].

Several factors were found to be significantly associated with the DT. The median DT in our study was 667 min for multiparous women, which was must shorter than that in nulliparous women. Thus, parity has proved to be the main contributing factor for the DT [13], and an obvious reduction in DT was noted in multiparous women when compared with nulliparous women [14, 15]. Similar findings have been reported by Blankenship et al., who showed that the dilation time from 6 to 10 cm was 3.28 h in nulliparous women, which was significantly slower than the 2.03 h recorded for multiparous women [16].

Another finding of the current study is that the state of the membranes could significantly affect DT: PROM was found to be associated with a shorter time to delivery—a finding similar to that observed in other studies [17, 18]. In 2021, a randomized controlled trial was conducted by the Pittsburgh Medical center to compare early artificial rupture of membranes or expectant management in 160 patients who underwent cervical ripening with Foley catheter expulsion; in that study, the median DT was 8 h shorter for women who underwent early artificial rupture than in women with expectant management [19]. However, Devillard et al. compared a double balloon catheter with oxytocin versus a vaginal dinoprostone insert in women with PROM at term and found no significant difference in DT between the two groups [20]. An exploration of the mechanism for the decreased DT associated with PROM may be interesting, although we concluded that the method of IOL may not be a major contributing factor for this association.

Advanced maternal age was found to be associated with longer durations of both first- and second-stage labor. However, this difference was not significant in the case of women of age < 35 years in our study. A cohort study from Midwives Alliance of North America has shown that compared with multiparous women aged ≥ 35 years, those aged < 35 years could complete the active phase of labor in approximately 1 h less [21]. Advanced age has also been shown to be a major risk factor for increased risk of obstetric trauma [22, 23]. For each additional year of age past 18 years at first delivery, the risk of major pelvic floor trauma has been found to incrementally increase by a ratio of 1.064 over the risk from the previous year [24]. One possible explanation for these phenomena may be the reduction in the elasticity of tissue and bone density with advancing age [25, 26].

Regarding fetal macrosomia, most previous studies have focused on the increased risk of perinatal morbidity and mortality, including dystocia, cesarean delivery, postpartum hemorrhage, and facial nerve palsy [27, 28]. A multicenter cohort that included more than 110,000 women at term revealed that when the estimated fetal weight is > 3500 g, the odds of cesarean delivery were significantly raised, and that the highest odds of cesarean delivery were observed in women with gestational diabetes mellitus and an estimated fetal weight of  ≥ 4000 g [29]. However, it is difficult to accurately estimate fetal weight by clinical and ultrasound examination, which was confined when applied [30, 31]. Moreover, several studies have shown that IOL at or beyond 38 weeks for suspected fetal macrosomia is associated with a decrease in the rate of fetal fractures and DT [32]. We found that a fetal weight of > 4000 g was more liable to prolong labor, and suspicion regarding fetal macrosomia should be raised if the labor progress is unsatisfactory.

Before IOL, the cervix is traditionally evaluated using the Bishop score. However, this assessment tool has high inter- and inter-observer variability and relies on clinical assessment, which has poor predictive value [33, 34]. According to the protocol followed at our center, for women with an unfavorable cervix for IOL, we initiate IOL during the night-time so that delivery occurs during the daytime. This is to decrease the risk of neonatal mortality, which is associated with night-time delivery. Similarly, when the cervical conditions are favorable, IOL is traditionally initiated during the day. Studies have shown that compared with women who had night-time deliveries, those who gave birth during the daytime reported better childbirth experiences [35]. Therefore, we replaced Bishop score with placement time of the dinoprostone insert and found that the DT was shorter in women for whom the dinoprostone insert was placed during the day than in those for whom the insert was placed during the night. However, further studies are warranted to explore the effect of the circadian rhythm on DT.

Many studies have identified high BMI as an independent risk factor for vaginal delivery [36, 37]. A cohort study on > 5000 parturients has shown that compared to women with a BMI less than 30, obese women with a higher BMI had a longer duration and slower progression of the first stage of labor, although there were no differences in the timing of cervical dilation from 6 cm to complete dilation [38]. However, our study did not show any effect of BMI on DT. There may be several reasons for this. First, our study included only those women who completed vaginal delivery and excluded women who required conversion to cesarean delivery during labor. Second, most of the women in our study (88.1%) had BMI less than 25.

On the basis of these factors, a mathematical model was developed for the individualized prediction of DT following application of a dinoprostone vaginal insert. The model integrates the five significant variables that were found to influence DT, namely, multiparity, advanced maternal age, fetal macrosomia, daytime insertion of dinoprostone, and PROM. The model is given as follows: “*Y* = 804.478 − 125.284 × multiparity + 765.637 × age (≥ 35 years) + 411.511 × fetal macrosomia − 593.358 × daytime insertion of dinoprostone − 125.284 × PROM.” In recent years, several large-scale studies have shown that perinatal outcomes are poor for deliveries that occur outside the normal working hours [39, 40]. In particular, a retrospective cohort study of 1 million live births in Scottish has shown that the risk of neonatal death was 4.2 per 10,000 for deliveries that occurred during the daytime on a weekday and 5.6 per 10,000 for deliveries that occurred at other times [39]. To a certain extent, this model could be valuable both for obstetricians, by helping them take measures to minimize the risk in the maternity ward, and for patients, by providing them information about what they can expect during delivery and thereby enhance their childbirth experience.

The model developed in our study offers several advantages: (1) It allows for the application of a standardized equation for predicting the DT. (2) It enables the assessment of the effect of different factors on DT by dinoprostone-induced labor, rather than labor progress, with the former having the ability to reduce the risk factors in the ward. (3) It provides an objective assessment and quantification of DT on the basis of the individual clinical characteristics.

This study has a few limitations. Primary among them is the retrospective, single-center design. The model population comprised women from central China, who mainly belonged to the Han community. Furthermore, amniotomy and administration of oxytocin following removal of the insert is a routine practice adopted at the maternity wards at our center. In light of these points, our results might not be generalizable to other ethnic groups and do not represent labor progress in women managed with other methods of IOL.

To summarize, we have demonstrated that parity, maternal age, fetal macrosomia, the status of the membrane, and time of dinoprostone placement are closely associated with DT following IOL. On the basis of our findings, we developed an equation that would help estimate DT following use of dinoprostone. We believe that this equation could be an effective tool to help schedule deliveries according to the ward’s activity, which, in turn would be useful for obstetricians, midwives, and nurses.

## Data Availability

The datasets analyzed during the study are available from the corresponding author on reasonable request.
